# Multiple Applications of a Novel Cationic Gemini Surfactant: Anti-Microbial, Anti-Biofilm, Biocide, Salinity Corrosion Inhibitor, and Biofilm Dispersion (Part II)

**DOI:** 10.3390/molecules25061348

**Published:** 2020-03-16

**Authors:** A. Labena, M. A. Hegazy, Radwa M. Sami, Wael N. Hozzein

**Affiliations:** 1 Egyptian Petroleum Research Institute (EPRI), Nasr, Cairo 11727, Egypt; mohamedhegazy997@gmail.com (M.A.H.); radwa_samii@yahoo.com (R.M.S.); 2 Bioproducts Research Chair, Zoology Department, College of Science, King Saud University, Riyadh 11451, Saudi Arabia; hozzein29@yahoo.com; 3 Botany and Microbiology Department, Faculty of Science, Beni-Suef University, Beni-Suef 62511, Egypt

**Keywords:** cationic gemini surfactant, mild steel, biocidal activity, corrosion inhibitor, sulfidogenic bacteria, anti-biofilm, bio-dispersion agent

## Abstract

The Egyptian petroleum industries are incurring severe problems with corrosion, particularly corrosion that is induced by sulfidogenic microbial activities in harsh salinity environments despite extensively using biocides and metal corrosion inhibitors. Therefore, in this study, a synthesized cationic gemini surfactant (SCGS) was tested as a broad-spectrum antimicrobial, anti-bacterial, anti-candida, anti-fungal, anti-biofilm (anti-adhesive), and bio-dispersion agent. The SCGS was evaluated as a biocide against environmental sulfidogenic-bacteria and as a corrosion inhibitor for a high salinity cultivated medium. The SCGS displayed wide spectrum antimicrobial activity with minimum bactericidal/fungicidal inhibitory concentrations. The SCGS demonstrated anti-bacterial, anti-biofilm, and bio-dispersion activity. The SCGS exhibited bactericidal activity against environmental sulfidogenic bacteria and the highest corrosion inhibition efficiency of 93.8% at 5 mM. Additionally, the SCGS demonstrated bio-dispersion activity against the environmental sulfidogenic bacteria at 5.49% salinity. In conclusion, this study provides a novel synthesized cationic surfactant with many applications in the oil and gas industry: as broad-spectrum antimicrobial and anti-biofilm agents, corrosion inhibition for high salinity, biocides for environmentally sulfidogenic bacteria, and as bio-dispersion agents.

## 1. Introduction

The oil and gas industries are suffering from many corrosion problems that are induced by high salinity corrosive environments and sulfidogenic microbial activities, in bulk phases and on metal surfaces, although corrosion inhibitors and biocides are extensively used. Designing a novel cationic surfactant with specific physicochemical properties, multifunctional groups, and multiple purposes has attracted the attention of scientists. Cationic surfactants (in an aqueous media) possess high surface-active properties, and their hydrophilic parts carry positive charges. Cationic gemini surfactants are considered to be a new class of cationic surfactant, as they consist of two identical cationic surfactants, i.e., two identical hydrophilic head-groups and two hydrophobic tail-groups that are separated by a covalent spacer [1,2]. Gemini surfactants exhibit higher surface-active properties, a lower critical micelle concentration (*CMC*_c_), better foaming, better wetting, and stronger anti-microbial (with a much broader spectrum) and anti-adhesive activities as compared to the corresponding monomeric surfactants [3,4,5]. However, they display lower biodegradability properties than monomeric surfactants.

The activity of cationic gemini surfactants against microorganisms in bulk phases and on surfaces (biofilms) generally depends on their structures [6]. The antimicrobial activity in the bulk phases mainly depends on two quaternary nitrogen atoms (R_4_N^+^), alky-chain lengths, counter ions, spacer structures, and the effect of additive functional groups, such as pyridine rings and azomethine [5,7,8,9]. The hypothesized antimicrobial activity mechanism of cationic gemini surfactants was attributed to the electrostatic interaction between the surfactant cationic group (R_4_N^+^) and the negatively charged group of the plasma membrane (lipoprotein) of bacteria. This leads to changes in the potentiality of the cell surface.

The hydrophobic chain of cationic gemini surfactants can lead to the penetration of the membrane of the microbial cell, which leads to a loss of the permeable selectivity of the cell and, consequently, the cell’s death [10]. Many microorganisms are able to form biofilms, which are difficult to eradicate with ordinary biocides as a consequence of their strong adhesion to surfaces and their high resistance to many antimicrobial agents. Biofilms are composed of layers of a microbial community, extracellular polymeric substances (EPS), inorganic materials, and water. The application of cationic gemini surfactants as anti-adhesive (anti-biofilm) agents was previously reported [5]. The anti-adhesive activity of cationic gemini surfactants was mainly attributed to their hydrophobicity, as these compounds display high surface-active properties that allow for them to coat or cover a surface via hydrophobic interactions [11].

There are several strategies for cationic gemini surfactant deposition on surfaces to reduce or prevent cell adhesion and biofilm development, such ion exchange, ion pairing, or hydrophobic interactions [12,13]. In the oil and gas sector, microbial adhesion produces many problems in the economy and environment in the form of microbially-influenced corrosion (MIC). MIC can cause effective increases in the maintenance costs and the degradation of the structural integrity with subsequent risks on platforms and even the loss of human life. Sulfidogenic bacteria or sulfate-reducing bacteria (SRB) have been repeatedly correlated with MIC. The sulfidogenic bacteria are known as an anaerobic bacterial group that can reduce sulfate (SO_4_^2−^) to sulfide (S^2−^).

The corrosiveness of such a microbial community is due to the produced metabolites (such as hydrogen sulfide), a cathodic depolarization process, and their microbial attachment to metal surfaces (as biofilms) [14]. The application of cationic gemini surfactants as corrosion inhibitors and biocides can afford many features, such as the separation or protection of metal surfaces from water and corrosive mediums (corrosive solutions and microbial-metabolites), which postpones the reduction and oxidation corrosion reactions and provides biocidal activity against MIC in bulk phases (against planktonic bacteria) and on metal surfaces (against microbial adhesion) [15].

Therefore, the objective of the present study was to evaluate a novel synthesized cationic gemini surfactant as a wide-spectrum antimicrobial agent and as an anti-bacterial and anti-biofilm (anti-adhesive) agent against standard aerobic bacterial cells in the bulk phase and the surface, respectively. The novel cationic gemini surfactant was evaluated as a biocide and as a corrosion inhibitor against environmental sulfidogenic bacteria, which were collected from an infected water tank with a salinity of 5.49% NaCl. The synthesized cationic gemini surfactant (SCGS) was evaluated as a bio-dispersion agent against the environmental sulfidogenic bacteria.

## 2. Results and Discussion

**Figure 1:**
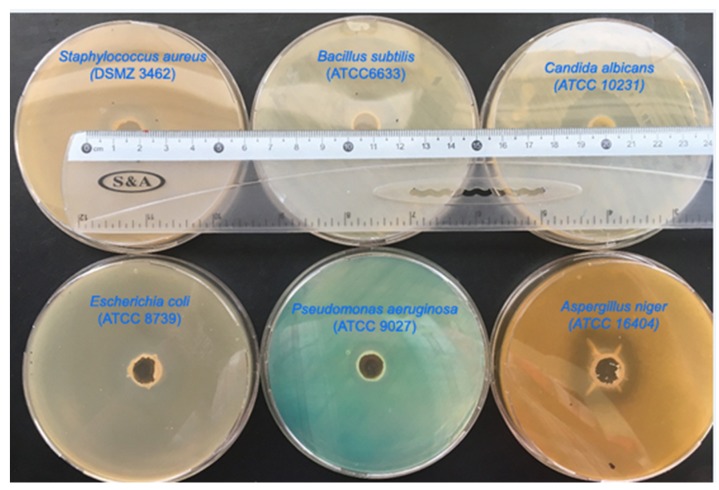
The antimicrobial activity of the SCGS using a modified agar well diffusion method against (a) strains *Staphylococcus aureus* (DSMZ 3463), *Bacillus subtilis* (ATCC 6633), *Escherichia coli* (ATCC 8739), *Pseudomonas aeruginosa* (ATCC 9027), *Candida albicans* (ATCC 10231), and *Aspergillus niger* (ATCC 16404).

In the present work, the SCGS [16] was applied as a broad antimicrobial agent against standard microbial strains. The results that are shown in Table 1 and Figure 1 represent a broad antimicrobial activity of the SCGS with zone inhibitions ranging from 20–30 mm for the bacterial isolates and 28–33 mm for the yeast and the fungal strains, respectively, in comparison with the positive control antimicrobial agent. The SCGS displayed higher antibacterial efficiency against the Gram-positive bacteria (29–32 mm) as compared with the Gram-negative bacteria (20–22 mm). This difference in susceptibility is presumably attributed to the differences in the cytoplasmic membrane physiology of the two bacterial types, as previously explained [17,18].

**Figure 2:**
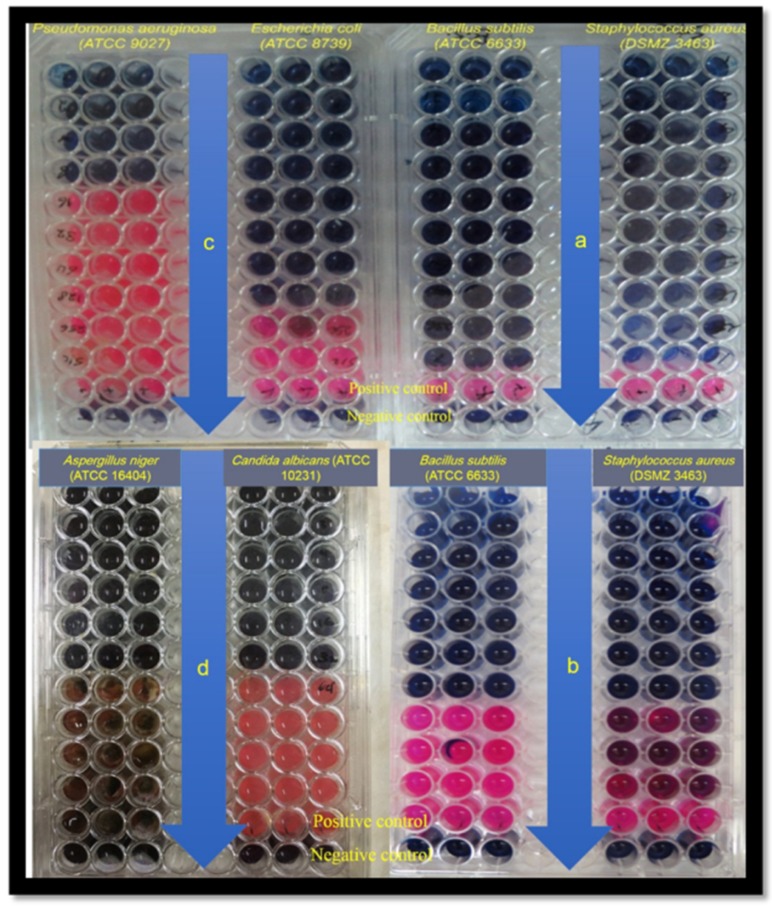
Minimum inhibitory (MIC) and minimum bactericidal/fungicidal (MBC/MFC) concentrations of the SCGS estimation using a two-fold micro dilution method in 96 well micro-titer plates in comparison to a positive control (inoculated with microorganism without the SCGS) and a negative control (only sterile media). The plates were visually elaborated using a resazurin as an oxidation-reduction indicator at a concentration of 0.015%. The well with no color change (blue resazurin) means negative result or no growth however the changed color (pink color) means positive result or there is bacterial growth. (a, b) Were the plates of *Staphylococcus aureus* (DSMZ 3463), *Bacillus subtilis* (ATCC 6633) with more dilution. (c) The plate of *Escherichia coli* (ATCC 8739), *Pseudomonas aeruginosa* (ATCC 9027). (d) The plate of *Candida albicans* (ATCC 10231) and *Aspergillus niger* (ATCC 16404).

The SCGS displayed MIC and MBC (0.004–0.02 mM and 0.009–0.02 mM, respectively) for Gram-positive bacteria and (0.04–0.62 mM and 0.04–0.31 mM, respectively) for Gram-negative bacteria. In addition, the SCGS showed anti-fungal activity against standard yeast and fungal strains (16–17 mm) with MIC/MFC (0.02 and 0.04 mM) for the candida strain and (0.3 and 0.3 mM) for the fungal strain (see Table 2, Figure 2).

Many researchers reported the antimicrobial activity of synthesized cationic surfactants that have 10 or 12 carbon atoms within an alkyl chain [19,20,21]. Increases in the antimicrobial activity were associated with alkyl chain elongation [22]. The supposed interpretation of the SCGS antibacterial activity was attributed to an electrostatic interaction between the positive ammonium group, R_4_N_+_ of the SCGS, and the negatively charged lipoprotein of the bacterial cell membrane, which leads to cell disruption [23]. In addition, the hydrophobic chain of the SCGS easily penetrated the microbial cell membrane, which led to damage of the cell’s selective permeability and, consequently, the death of the cells [10].

Another possible hypothesized mechanism of the SCGS antimicrobial activity is an influx of molecules of the surfactant into the cell leading to interactions with particular organelles (such as the mitochondria and vacuoles) [24]. The fungicidal activity of the SCGS was attributed to its ability to incorporate the plasma membrane, which leads to its dysfunction [25]. SCGS was previously reported to attach to the cell surface of fungal cells and reverse the membrane charge from negative to positive [26,27]. It was reported that pyridine-based gemini surfactants cause pore formation on the plasma membranes of fungal cells, leading to the dysfunction of the cells. The application of pyridine-based gemini surfactants on fungal cells caused increases in the reactive oxygen species (ROS). Therefore, the surfactant easily penetrated the cell and interacted with the membrane of the mitochondria, which led to severe oxidative stress [21].

**Figure 3:**
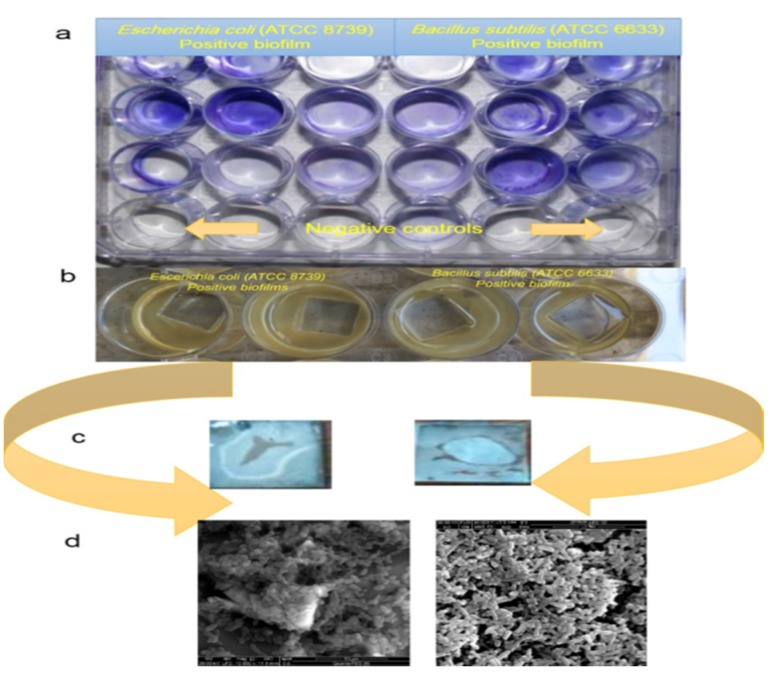
Positive induced bacterial biofilms of *Bacillus subtilis* (ATCC 6633) and *Escherichia coli* (ATCC 8739) on a 24 dilution titer plates. (**a**) the cultivated biofilms on the plate surface, (**b**) the cultivated biofilms on the glass surface (1.0 × 1.0 × 0.3 cm), (**c**) the dried biofilms on the glass surface, (**d**) the scanning electron microscopy (SEM) images of *Bacillus subtilis* (ATCC 6633) (right side) and *Escherichia coli* (ATCC 8739) (left side).

The first step in microbial cell-related infections is their surface adhesion ability. The transformation process of planktonic cells (in bulk phase) to sessile cells (on a surface—called biofilms) has been associated with increased levels of antimicrobial agent resistance. In many circumstances microbial adhesion is driven by flagellar proteins, the secretion of an extracellular polymeric substance (EPS) (which is composed of polysaccharides, lipids, proteins, etc.), mass transportation, electrostatic interactions, Van der Walls forces, hydrophobicity, hydrogen bonding, and the liquid flow rate [28]. It was reported that, once a biofilm is formed on a surface, it is difficult to inhibit and/or eradicate by normal antimicrobial agents [29]. Therefore, one of the most notable aims of this research was to investigate the possible application of the SCGS as anti-bacteria anti-biofilm (anti-adhesive) agents and as bio-dispersion agents (Figure 3).

The results that are presented in Table 3 showed that the SCGS displayed anti-biofilm activity toward *B. subtilis* and *E. coli* induced biofilms with MBICs of 0.31 and 0.62 mM for Gram-positive and Gram-negative bacteria-induced biofilms, respectively. The SCGS displayed bio-dispersion activity toward the positively developed biofilms with minimum biofilm eradication concentrations (MBECs) of 0.31 and 0.62 mM for Gram-positive and Gram-negative bacteria-developed biofilms, respectively (Table 3). The explanation of the anti-adhesive activity of the SCGS against the Gram-positive and Gram-negative bacterial developed biofilms were attributed to its hydrophobicity, as this compound coated or covered the plate surface via hydrophobic interaction, as previously reported [30]. It was reported that the bacterial cell adhesion to surfaces is the first step of biofilm development and this process not only relies on the cell envelope properties, such as hydrophobicity or roughness, but also on special substratum properties [31]. There are several strategies of cationic surfactant deposition on surfaces, such as ion exchange, ion pairing, or hydrophobic interactions, to reduce or prevent cell adhesion and biofilm development [12,13].

**Figure 4:**
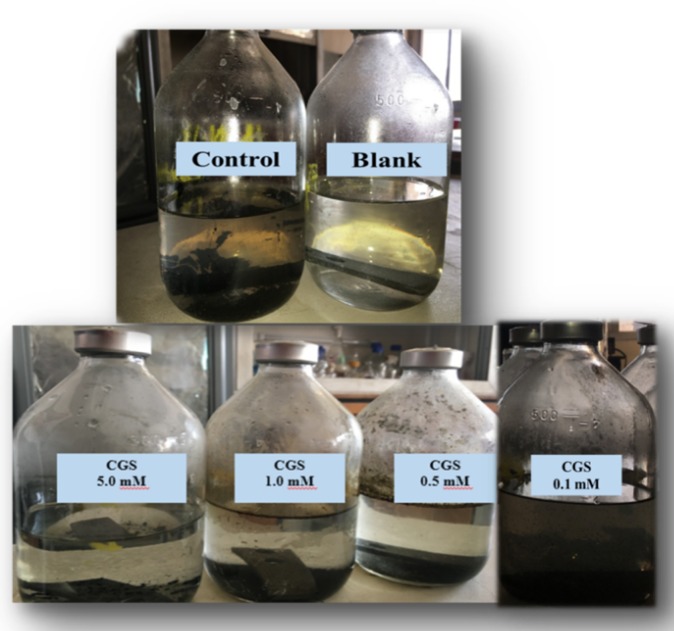
The application of the cationic gemini surfactant (SCGS) at different concentrations (0.1, 0.5, 1.0, 5.0 mM) in comparison to control reactor (inoculated with enriched environmental sulfidogenic bacteria at a salinity of 5.49% NaCl), and blank reactor (un-inoculated with enriched environmental sulfidogenic bacteria at a salinity of 5.49% NaCl).

The application of cationic gemini surfactants in the petroleum sector as a biocide and a corrosion inhibitor has attracted the attention of scientists [32,33]. Gemini surfactants display a strong metal protection activity in comparison to their monomeric counterparts, as the gemini surfactants possess a significant low critical micelle concentration (*CMC*c), a spacer type induced efficiency, and high hydrophobicity and high adhesion properties [34]. Furthermore, cationic gemini surfactants possess a strong biocidal activity, not only against aerobic bacteria but also against anaerobic bacteria in the bulk phase and on metal surfaces, which is attributed to their strong electrostatic interaction and physical disruption. Therefore, the present work aimed to apply the SCGS as a biocide against environmental sulfidogenic bacterial communities cultivated at high salinity (5.49% NaCl) and as a corrosion inhibitor against a cultivated salinity medium when the bulk phase and the metal surfaces are totally free from the cultivated bacteria (see Figure 4).

Table 4 shows that the highest metal corrosion rate (0.69 g/m^2^ d) of the blank reactor (absent of the enriched bacteria) when compared with the metal corrosion rate (0.31 g/m^2^ d) of the control reactor (in the presence of the enriched bacteria). The harmful effect of the chloride anions on the metal surface is the explanation for this result [35]. The harmful chloride anions strongly penetrated the oxide films that developed on the metal surface through the pores and then through colloidal dispersion. Another explanation of this effect is the adsorption behavior of the chloride anion, such as when the metal surface was covered with chloride anions; this promotes the hydration of the metal ions and, hence, sustains the pit and crevice corrosion [36].

In this reaction, the iron-chloride anion serves as the catalyst for further metal corrosion [37]. The lowest metal corrosion rate (0.31 g/m^2^ d) of the control reactor (in the presence of enriched sulfidogenic bacteria) in comparison to the metal corrosion rate (0.69 g/m^2^ d) of the blank reactor (in the absence of enriched sulfidogenic bacteria) was accredited to the effect of the sulfidogenic bacterial biofilm that covered and protected the surface of the metal from the corrosive and harmful chloride anion effects [38,39]. The sulfidogenic bacteria metal corrosion rate was mainly attributed to their activity in the bulk phase, as previously reported by Von Wolzogen Kuhr and Van der Vlugt [40].

Sulfidogenic biofilms induce severe localized corrosion in comparison to their planktonic SRB counterpart via their ability to entrap and localize the corrosive sulfidogenic metabolites on the metal surface. In addition, increases of the adhered cells on the metal surface, in the form of a biofilm, mainly depend on the excessive electrons that are induced by the cathodic depolarization source. In this respect, these electrons can be used as electron donors by sulfidogenic biofilms in their metabolites when other electron donors are not present [41]. The corrosion rates of the metal were gradually reduced when the SCGS was applied at different concentrations. The lowest corrosion rate was achieved at a concentration of 5 mM with a metal corrosion inhibition efficiency of 93.8% (see Table 4, Figure 4). The SCGS showed a biocidal effect on the sulfidogenic bacteria at concentrations of 0.5, 1, and 5 mM. The MBIC of the SCGS was attributed to a concentration of 0.5 mM, which visually did not show any developed biofilms on the metal coupons.

**Figure 5:**
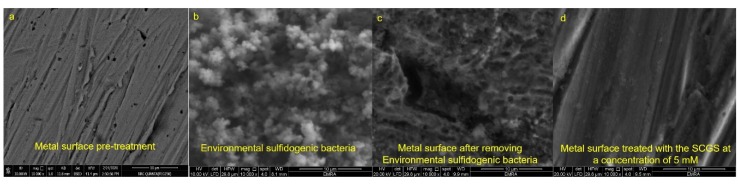
SEM images (**a**) the cleaned metal surface, (**b**) The sulfidogenic biofilm, (**c**) the metal surface (at a salinity of 5.49% NaCl) after removing the biofilm, and (**d**) the environmental sulfidogenic bacteria inoculated with 5 mM SCGS. Scale bar = 10 µm.

The obtained results were confirmed while using SEM analysis of the cleaned metal surface, the cultivated sulfidogenic bacterial reactor, the metal coupon after scratching the developed biofilms, and the coupon with the highest metal corrosion rate inhibition efficiency (5 mM SCGS) (Figure 5).

**Figure 6:**
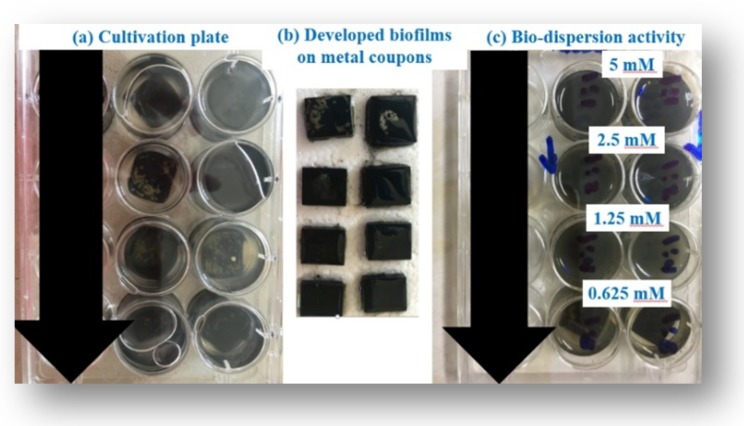
The bio dispersion activity of the SCGS on the metal surface against the environmental sulfidogenic bacteria at 5.49% salinity. (**a**) the cultivated sample (**b**) the coupons after washing (**c**) the bio-dispersion activity of the SCGS with concentration of 5, 2.5, 1.25, and 0.625 mM. The figure showed that the concentration of 1.25 mM is the MBEC of the SCGS on the metal surface against the environmental sulfidogenic bacteria at 5.49% salinity.

The SCGS displayed bio-dispersion power against the developed sulfidogenic bacterial biofilms after two weeks of cultivation with an MBEC of 0.625 mM that was visually observed from the absence of relative changes in the bulk phase turbidity in comparison to the high concentrations of 5, 2.5, and 1.25 mM (Figure 6 and Table 5).

The inhibitory mechanism of action of the applied SCGS on a metal surface, in the appearance of the environmental sulfidogenic bacteria cultivated in a high salinity medium (5.49% NaCl), could be attributed to its chemical structure and adsorption properties. It was previously reported that the inhibiting mechanism of cationic surfactants is related to their adsorption and the formation of protective layers at the metal/liquid interface [42]. There are two adsorption types that may occur on a metal surface: physical and chemical adsorption. Physical adsorption is induced via an electrostatic attraction between the group carrying a charge and the charge of the metal surface. However, the chemical adsorption might take place via charge sharing between unshared electron pairs (lone-pair) in the surfactant molecule and the metal surface [43].

The applied SCGS adsorbed on the metal surface is supported by its two quaternary nitrogen atoms (R_4_N^+^) at the cathodic site and two counter ions (Br^-^), the π-electrons of two pyridine rings, and two azomethine (–CH=N–) groups at the anodic site. The adsorption modes of gemini surfactants depend on their concentrations in the solution and on the surface. At a lower concentration, the adsorption occurs via the binding of the gemini surfactants horizontally to the hydrophobic region. At higher concentrations, the adsorption of surfactant occurs perpendicularly until the surface is completely saturated with the surfactant. The biocidal effect of the SCGS was credited to the effect of its structure, two quaternary nitrogen atoms (R_4_N^+^) at the cathodic site, and two counter ions (Br^−^), the π-electrons of two pyridine rings, and two azomethine (–CH=N–) groups at the anodic site [7,8,9,44,45,46,47].

A comparative study was conducted concerning the antimicrobial activity (against Gram-positive, Gram-negative bacteria, candida, and fungi), minimum inhibitory concentration (MIC), minimum bactericidal concentration (MBC), minimum fungicidal concentration (MFC), minimum biofilm inhibitory concentration (MBIC), minimum biofilms eradication concentration (MBEC), and corrosion inhibition efficiency (IE) to visualize the performance and the efficiency of the present synthesized surfactant in comparison with other synthesized surfactants (Table 6).

## 3. Materials and Methods

### 3.1. The Synthesized Cationic Gemini Surfactant (SCGS)

**Figure 7:**
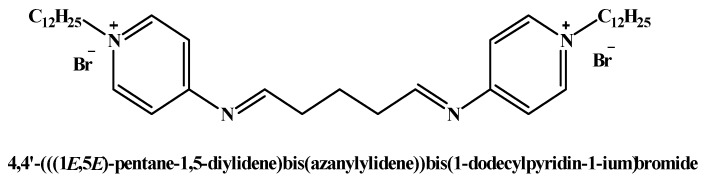
The chemical structure of the synthesized cationic gemini surfactant, 4,4′-(((1E,5E)-pentane-1,5-diylidene)bis(azanylylidene))bis(1-dodecylpyridin -1-ium) bromide [16]. The synthesis had a total yield of 91.3% (for more details see the supplementary materials).

We successfully synthesized and characterized the cationic surfactant (Figure 7) [16].

### 3.2. Application of the SCGS as a Broad Antimicrobial, Anti-Bacterial, Anti-Biofilm, Bio-Dispersion Agent

#### 3.2.1. Microbial Strains

The following strains, *Staphylococcus aureus* (DSMZ 3463), *Bacillus subtilis* (ATCC 6633), *Escherichia coli* (ATCC 8739), *Pseudomonas aeruginosa* (ATCC 9027), *Candida albicans* (ATCC 10231), and *Aspergillus niger* (ATCC 16404), were used in this study as standard microbial strains.

#### 3.2.2. Cultivation Conditions

The bacterial isolates were sub-cultured on trypticase soy broth (TSB) or trypticase soy agar (TSA) (Difco Co; Becton Dickinson, Sparks Glencoe, MD, USA) at 37 °C for an incubation period of 24 h. The yeast and fungal strains were sub-cultured on Sabouraud dextrose broth (SDB) or Sabouraud dextrose agar (SDA) (Difco Co; Becton Dickinson, Sparks Glencoe, MD, USA) at 30 °C for an incubation period of 48 h.

#### 3.2.3. Anti-Microbial Activity

In this work, the biological activity of the SCGS was estimated using the agar well diffusion method, as previously reported [55]. The tested microbial strains were streaked on the agar plates, and then the surfaces were cut into 10 mm wells using a sterile borer. We introduced 100 µL of the SCGS into each well. The biological activity was evaluated via measuring the clearing zones at the end of the incubation period (overnight at a temperature of 37 °C for the bacteria and for 48 h at a temperature of 30 °C for the yeast and fungi). The test was performed three times, and the average values were recorded. Sterile water was used as a negative control, and amoxicillin (100 ppm), tetracycline (100 ppm), fluconazole (100), and benzalkonium chloride (100 ppm) were used as the positive controls.

#### 3.2.4. Minimum Inhibitory (MIC) and Minimum Bactericidal/Fungicidal (MBC/MFC) Concentrations

The minimum inhibitory (MIC) and minimum bactericidal/fungicidal (MBC/MFC) concentrations of the SCGS were determined using a two-fold micro dilution method in 96-well micro-titer plates with modifications [56]. The bacteria, yeast, and fungal strain inocula were prepared according to the Clinical Laboratory Standards Institute (CLSI) method [57,58]. We serially diluted 100 µL of the SCGS (TSB and SDB for the bacterial strains and the yeast and the fungal strains, respectively) onto the micro-titer plates and then further inoculated them with 100 µL of the microbial inocula parallel with a positive control (inoculated without the SCGS) and a negative control (only sterile media). The micro-titer plates were then incubated under aerobic conditions for an incubation period of 20 h at 37 °C and 72 h at 30 °C for the bacteria and yeast/fungal strains, respectively. Three wells were performed for each test. The MIC was determined as the lowest concentration of the SCGS that inhibits the development of visible bacterial, yeast, and fungal growth on cultivated media after an incubation period. At the end of the incubation, resazurin was added to the wells (30 µL each) as an oxidation-reduction indicator at a concentration of 0.015%, and they were further incubated for 2 h at 37 °C for the visual observation of color change. A well with no color change (blue resazurin) indicated a negative result or no growth resazurin, and a changed color (pink color) indicated a positive result or bacterial growth.

In order to estimate the minimum bactericidal/fungicidal concentrations (MBC/MFC) of the SCGS needed to indicate 99.5% killing of the original inoculum, before adding the resazurin indicator, 10 µL was taken from the wells with no observed growth and further sub-cultured onto plates of agar of their related specific media [59].

#### 3.2.5. Anti-Microbial Biofilms and the Minimum Biofilm Inhibitory Concentrations (MBICs)

A semi-quantitative adherence assay on 96-well tissue culture plates was used to study the effects of the SCGS on the developed bacterial biofilms (*Bacillus subtilis* (ATCC 6633) and *Escherichia coli* (ATCC 8739), as previously reported [60] with minor modifications. Briefly, fresh overnight inocula of the bacterial strains were prepared according to the CLSI method [57]. We serially diluted 100 µL of the SCGS while using TSB (that was supplemented with 1% Glucose) onto the micro-titer plates. The micro-titer plates were inoculated with 100 µL of the freshly prepared inocula. The test was performed in parallel with positive (an inoculated well without the SCGS) and negative (only media) controls. After the incubation period (20 h at 37 °C temperature) ended, the plates were cleaned three times at pH 7.4 with 200 μL of 1× phosphate buffer saline (PBS), dried, fixed with ethanol, and then stained with crystal violet (0.1%). After staining for 10 min., the wells were washed again to remove the excess crystal violet stain and then dried at room temperature for 2 h. The developed bacterial biofilm appeared as purple rings that formed on the bottom and sides of the well. The minimum biofilm inhibitory concentration (MBIC) was calculated as the lowest concentration of the anti-biofilm agent (SCGS) that inhibits the development of visible microbial growth adherence (biofilm) on TSB (that was supplemented with 1% Glucose) after an incubation period.

#### 3.2.6. Bio-Dispersion Activity and Minimum Biofilm Eradication Concentration (MBEC)

The SCGS was tested for its ability to disrupt or eradicate (as a bio-dispersion agent) against the well-developed bacterial biofilms. In this respect, the developed bacterial biofilms (on the glass surface (1.0 × 1.0 × 0.3 cm) in the micro-titer plates after the 20 h incubation period) were washed twice with PBS (pH 7.4) to remove the unattached bacterial cells. Subsequently, we serially diluted 100 µL of the SCGS onto the micro-titer plates with the developed bacterial biofilms and incubated the plates for 2 h. The detailed minimum biofilm eradication concentration (MBEC) was obtained after washing, fixing, and staining, as above.

### 3.3. SCGS as a Biocide Against Environmental Sulfidogenic-Bacteria and a Corrosion Inhibitor for Cultivated Medium–High Salinity

#### 3.3.1. Environmental Bacterial Community Source and Diversity

The environmental sample in this was obtained from a formation water tank from the Qarun Petroleum Company (QPC), Egypt, with a salinity of 5.49% NaCl. The sample was enriched many times and characterized using dissimilatory sulfite reductase-β subunit (dsrβ) that was based on denaturing gradient gel electrophoresis (DGGE), as previously reported [61]. The *Desulfovibrio* genus (phylum Proteobacteria, class Delta-proteobacteria) was the most frequently detected sulfidogenic bacteria. There was no detection of *Archaea* with the *dsrβ* gene in the DGGE band sequences.

#### 3.3.2. Cultivation Conditions and Experimental Design

The batch experiments were displayed using a modified Postgate’s C medium that we prepared anaerobically for the evaluation of the SCGS effect on environmental sulfidogenic bacteria with a cultivated salinity of 5.49% (NaCl), as previously reported [62]. The inocula for the inhibition experiment were enriched using a modified Postgate’s B medium (with the cultivated salinity) for 14 days at 37 °C [62]. The bacterial count was determined using the most probable number (MPN) method of 0.93 × 10^−6^ [63]. The SCGS reactors were established while using mild steel coupons with the chemical composition, as represented in Table 7 (AISI 1018 mild carbon steel strip measuring 2-7/8” × 7/8” × 1/8” (7.3 × 2.2 × 0.32 cm), COSASCO’s, Rohrback Cosasco Systems, Inc). The experiment was determined using different concentrations of the SCGS. Two experiments were performed in parallel: (i) the blank reactor experiment (un-inoculated modified Postgate’s C medium) and (ii) the control reactor experiment (inoculated modified Postgate’s C medium). A separate reactor was implemented for the sulfidogenic biofilm examination, and the metal surface after scratching the cultivated attached biofilm, and the metal surface with the sulfidogenic bacteria at an optimum SCGS concentration was examined while using scanning electron microscopy (SEM).

#### 3.3.3. Corrosion Inhibition and Biocidal Activity

The tested mild steel coupons were removed from the applied reactors at the end of the experiment and then immersed in a Clarke solution (1 L 36% HCl, 20 g Sb_2_O_3_, and 50 g SnCl_2_) for 10–15 s. After that, the tested mild steel coupons were cleaned with deionized water; afterwards, the coupons were cleaned with ethanol and finally kept dried. The weight loss was estimated (comparing the weight of the mild steel coupons before and after the experiment). From the weight loss results, the corrosion rate (g/m^2^ d) and the inhibition efficiency (%) of the metal corrosion were determined [64]. This experiment was duplicated. We used model Quanta 250 field emission gun (FEG) at magnification ranging from 14× up to 1,000,000×, the resolution for the gun was 1n, and the gun was operated at an acceleration voltage of 30 KV to confirm the efficiency of the SCGS as a biocide against the environmental sulfidogenic-bacteria in high salinity SEM (FEI company, Netherlands). The mild steel coupons (2.0 × 2.2 × 0.32 cm) of the cleaned surface cultivated a biofilm. After scratching the developed biofilm and then treating the biofilm with the optimum concentration of the SCGS, the coupons were taken from the reactors and first washed with phosphate buffer saline (pH 7.4) for 5 min. The mild steel coupons were fixed with 4% glutaraldehyde for 4 h, washed again with PBS (5 min. two times), and then washed out with distilled water twice (5 min. each). Next, the surfaces were dehydrated using different concentrations of ethanol (25%, 50%, 75%, and 100%) for 15 min. each and then kept dried in a desiccator for the SEM analysis.

#### 3.3.4. Bio-Dispersion Activity and Minimum Biofilm Eradication Concentration (MBEC)

To increase the applicability of the SCGS, it was evaluated as a bio-dispersion agent, not only against aerobic bacterial single strains but also against environmental anaerobic sulfidogenic bacterial diversity. The experiment was established with anaerobic modified Postgate’s C medium in a 12-well plate. The medium was prepared, purged with nitrogen, autoclaved, and then 4 mL was distributed on the wells on the coupons (1.5 × 1.5 × 0.3 cm COSASCO’s, Rohrback Cosasco Systems, Inc.) in parallel, adding paraffin oil as a seal for the anaerobic cultures. Resazurin was added at a concentration of 0.015% as an oxidation-reduction indicator in order to check the medium for oxygen prevention. The medium was inoculated with the sulfidogenic bacteria and incubated for two weeks. Subsequently, the coupons were taken from the medium and washed two times with PBS (pH 7.4) to remove the unattached sulfidogenic bacterial cells. The coupons with well-developed biofilms were put in well-plates with different concentrations of SCGS and incubated for a further 2 h. The media were checked for turbidity. They changed to a blackish color. The minimum biofilm eradication concentration (MBEC) was obtained as the lowest concentration that showed relatively no changes in the media.

## 4. Conclusions

1. The cationic gemini surfactant was successfully applied as a broad anti-microbial agent against standard bacterial, yeast, and fungal strains.
2. The minimum inhibitory, bactericidal, and fungicidal concentrations were achieved for the tested SCGS.
3. The SCGS displayed anti-bacterial, anti-biofilm, and bio-dispersion activity, and the minimum biofilm inhibitory and the minimum biofilm eradication concentrations were obtained against both aerobic and anaerobic bacteria.
4. The applied SCGS demonstrated biocidal activity against the environmental sulfidogenic bacteria and it acted as a corrosion inhibitor against the environmental sulfidogenic bacteria cultivated in a corrosive high salinity medium. The SCGS achieved a metal corrosion inhibition efficiency of 93.8% at a concentration of 5 mM.
5. The SCGS demonstrated bio-dispersion activity against the sulfidogenic bacterial biofilms on the metal surface with an MBIC of 0.5 mM and MBEC of 0.625 mM.

## Figures and Tables

**Table 1:** The antimicrobial activity of the synthesized cationic gemini surfactant (SCGS). The result was described as a mean of the inhibition zones diameter (mm).

Samples	*Staphylococcus aureus (DSM 3463)*	*Bacillus subtilis (ATCC 6633)*	*Escherichia coli (ATCC 8739)*	*Pseudomonas aeruginosa (ATCC 9027)*	*Candida albicans (ATCC 10231)*	*Aspergillus niger (ATCC16404)*
	Inhibition zone (mm)					
SCGS	30	29	25	20	33	28
* AMC	23	18				
* TE			22	20		
* Flu					27	
* BZK						26

* AMC, amoxicillin, TE, Tetracycline, Flu, Fluconazole, BZK. Benzalkonium chloride (concentration of 100 ppm).

**Table 2:** The minimum inhibitory concentration (MIC), the minimum bactericidal concentration (MBC), and the minimum fungicidal concentration (MFC) of the SCGS against different standard microbial strains. The result was represented as the mean of the samples concentrations (mM) with zero standard deviations (SD).

Sample	*Staphylococcus aureus* (DSM 3463)		*Bacillus subtilis* (ATCC 6633)		*Escherichia coli* (ATCC 8739)		*Pseudomonas aeruginosa* (ATCC 9027)		*Candida albicans* (ATCC 10231)		*Aspergillus niger* (ATCC16404)	
	MIC (mM)	MBC (mM)	MIC (mM)	MBC (mM)	MIC (mM)	MBC (mM)	MIC (mM)	MBC (mM)	MIC (mM)	MFC (mM)	MIC (mM)	MFC (mM)
SCGS	0.004	0.009	0.004	0.02	0.04	0.04	0.62	0.31	0.15	0.15	0.31	0.31

**Table 3:** The minimum biofilm inhibitory concentration (MBIC) and minimum biofilm eradication concentration of the SCGS against different standard developed bacterial biofilms. The result was represented as the mean of the sample concentrations (mM) with zero standard deviations (SD).

*Bacillus subtilis* (ATCC 6633)		*Escherichia coli* (ATCC 8739)	
MBIC (mM)	MBEC (mM)	MBIC (mM)	MBEC (mM)
0.31	0.31	0.62	0.62

**Table 4:** Metal corrosion rate (g/m^2^ d) and metal corrosion inhibition efficiency (%) of the SCGS reactors at different concentrations (mM) and inoculated with enriched environmental sulfidogenic bacteria cultivated at high medium salinity of 5.49% NaCl in comparison to a blank reactor (un-inoculated with the sulfidogenic bacteria with high medium salinity) and a control reactor (inoculated with the sulfidogenic bacteria with high medium salinity).

Cultivated Reactors	Metal Corrosion Rate (g/m^2^ d)	Metal Corrosion Inhibition Efficiency (%)
Blank	0.682 ± 0.02	0.0
Control	0.326 ± 0.07	52.9
0.1 mM	0.462 ± 0.1	32.3
0.5 mM	0.175 ± 0.005	75.0
1.0 mM	0.084 ± 0.007	88.2
5.0 mM	0.042 ± 0.004	93.8

**Table 5:** The minimum biofilm inhibitory concentration (MBIC) and minimum biofilm eradication concentration of the SCGS against sulfidogenic diversity developed bacterial biofilms. The result was represented as the mean of the sample concentrations (mM) with zero standard deviations (SD).

MBIC (mM)	MBEC (mM)
0.5	0.625

**Table 6:** Comparison of the biological activity and the corrosion inhibition efficiency between the synthesized surfactant and the other published synthesized surfactants.

Surfactants	* Test	** Unit	Biological Activity			Anaerobic Bacteria	Media	References
4,4′-(((1*E*,5*E*)-pentane-1,5-diylidene)bis(azanylylidene))bis(1-dodecylpyridin-1-ium) bromide			G + Ve	G − Ve	Candida & Fungi			
	DWD	mm	29–30	20–25	28–33	-	-	Present study
	MIC	mM	0.004	0.004–0.62	0.15–0.31	0.1	-	
	MBC or MFC	mM	0.009–0.02	0.04–0.031	0.15–0.31	0.5	-	
	MBIC	mM	0.31	0.62	-	0.5	-	
	MREC	mM	0.31	0.62	-	0.65	-	
	* IE	%	-	-	-	93.8	SRB and salinity	
hexamethylene-1,6-bis(*N*,*N*-dimethyl-*N*-dodecyldode cylammoniumbromide) (12-6-12).	MIC	mM				0.18		[48]

	IE	%				96	SRB and salinity	
ethane-1,2-diyl bis(*N*, *N*-dimethyl-*N*-alkylammoniumacetoxy) dichlorides (m-E2-m,m = 12, 14, 16)	DWD	mm	11–19	10–11	18–21			[49]
*N*1,*N*2-bis(2-(3-(4-(dodecanoyloxy) phenyl)propanamido)ethyl)-*N*1,*N*2,*N*2-tetramethylethane-1,2-diaminimum bromide (**AG12**), *N*1, *N*2-*bis*(2-(3-(4-(tetradecanoyloxy) phenyl)propanamido)ethyl)*N*1,*N*2,*N*2-tetramethyl ethane-1,2diaminimum bromide (**AG14**), *N*1, *N*2-bis(2-(3-(4(hexadecanoyloxy) phenyl) propanamido)ethyl) *N*1,*N*2,*N*2-tetramethyl ethane-1,2-diaminimum bromide (**AG16**)	DWD	mm	10–18	8–23	12–23	-		[50]
*N*-(2-(2-hydroxy ethoxy) ethyl)-*N*,*N*-dimethyloctan-1-aminium bromide (**HEDOB**), *N*-(2-(2-hydroxy ethoxy) ethyl)-*N*,*N*-dimethyldodecan-1-aminium bromide (**HEDDB**), *N*-(2-(2-hydroxy ethoxy) ethyl)-*N*,*N*-dimethylhexadecan-1-aminium bromide (**HEDHB**)	DWD	mm	15–22	16–22	13–15			[51]
	MIC	mM				0.5		
C_n_H_2n+1_OOCCH_2_N^+^(CH_2_)_2_-(CH_2_)_3_-NHOC-(CH_2_)m-CONH-(CH_2_)_3_-N+(CH_2_)_2_CH_2_COOC_n_H_2n+1_ (with *n* = 8, 10, 12 and *m* = 2, 3, 4),	MIC	mM	0.064–0.512	0.032–0.512	-	-	-	[52]
*N*-iso propyl *N,N*-dimethyl dodecan-1-aminium hydroxide	DWD	mm	35.5	20.75–25.75	-	-	-	[53]
	MIC	mM	0.1	1.0	-	1.0	-	
	MBC	mM	0.1	1.0	-	1.0	-	
	MBIC	mM	-	-	-	1.0	-	
	IE	%				92.0	SRB and salinity	
*bis (N*-ethyl-*N,N*-dimethyl dodecan-1-aminium hydroxide) phthalate	DWD	mm	37.5	25.5–28.0	-			[53]
	MIC	mM	0.1	1.0	-	1.0	-	
	MBC	mM	0.1	1.0	-	1.0	-	
	MBIC	mM				1.0	-	
	IE	%				94	SRB and salinity	
didecyldimethylammonium chloride (DDAC)	MIC	mM	-	-	-	1.3		[54]
	IE	%	-	-	-	91.4	SRB and salinity	

* Test: DWD, diffusion well diameter using the agar diffusion method; MIC is the minimum inhibitory concentration; MBC and MFC are the minimum bactericidal and fungicidal concentrations, respectively; MBIC is the minimum biofilm inhibitory concentration; MBEC is the minimum biofilm eradication concentration; and the IE is the inhibition efficiency. ** Unit: mm, millimeters, mM is millimoles.

**Table 7:** The chemical composition of a mild steel coupon AISI 1018 mild/low carbon steel strip COSASCO’s, Rohrback Cosasco Systems, Inc.

Element	Content
Carbon, C	0.14–0.20%
Iron, Fe	98.81−99.26% (as remainder)
Manganese, Mn	0.60–0.90%
Phosphorous, P	≤0.040%
Sulfur, S	≤0.050%

## Supplementary Materials

The following are available online, Figure S1. The chemical structure of the synthesized cationic gemini surfactant (SCGS) [16]. Figure S2. FTIR of the SCGS namely 4,4’-(((1E,5E)-pentane-1,5diylidene)bis (azanylylidene))bis(1-dodecylpyridin-1-ium) bromide [16]. Figure S3. 1H NMR of the SCGS namely 4,4’-(((1E,5E)-pentane-1,5-diylidene) bis(azanylylidene))bis(1-dodecylpyridin-1-ium) bromide [16].
