# Supplementary material for: Multiple Applications of a Novel Cationic Gemini Surfactant: Anti-Microbial, Anti-Biofilm, Biocide, Salinity Corrosion Inhibitor, and Biofilm Dispersion (Part II)
Source: Molecules. 2020 Mar 16;25(6):1348. doi: 10.3390/molecules25061348 (PMC7144103; doi:10.3390/molecules25061348)
Supplement: Supplementary file 1 [file molecules-25-01348-s001.pdf]

Materials and method:

Synthesis procedure:

The cationic gemini surfactant in the current research was prepared as follow. (i), a 0.1 mol of pyridin-4-amine was reacted with 5.006 g, 0.05 mol glutaraldehyde with using a solvent, ethanol, at 80°C for 6 h. Afterward, the mixture of the reaction was cooled and cleaned out using diethyl ether. The product was recrystallized by absolute ethanol. (ii) The product that obtained from the first step, "(1E,5E)-N1,N5-di(pyridin-4-yl)pentane-1,5-diimine, (5.046 g, 0.05 mol) pyridin-4-amine" allowed to react with (4.985 g, 0.05 mol) 1-bromododecane in the presence of ethanol as a solvent at 80 °C for 6 h . Afterward the mixture resulted from the reaction left to cool at room temperature and washed with diethyl ether. Furthermore, the product was recrystallized by absolute ethanol. Finally, the product namely, 4,4'-(((1E,5E)-pentane-1,5-diylidene)bis(azanylylidene)) bis (1-dodecylpyridin-1-ium) bromide was brown visages. The chemical structure of the product was shown in Figure 1. Further characterization of the synthesized cationic surfactant to the chemical structure was performed by FTIR and ^{1}HNMR spectroscopic analysis [16].

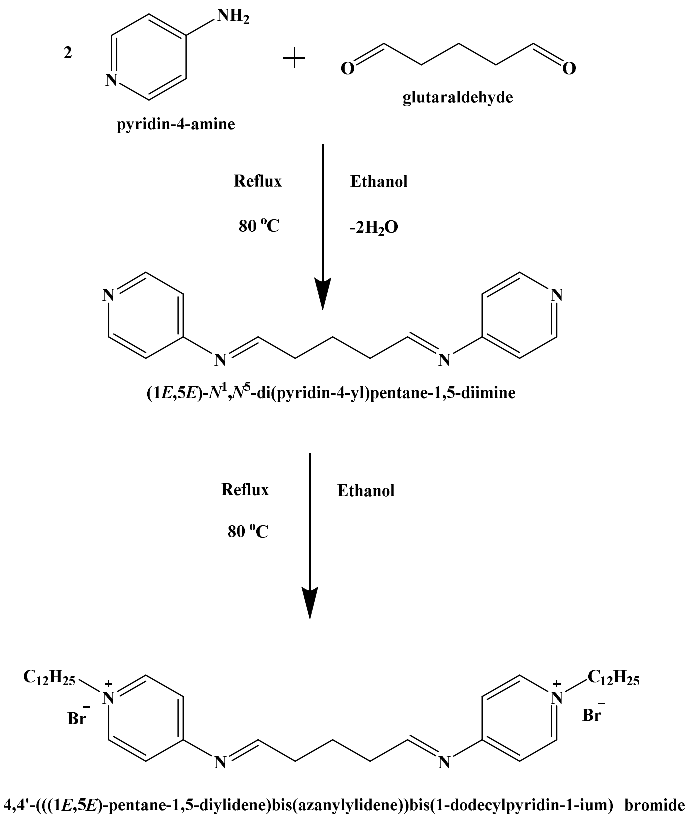

Figure S1. The chemical structure of the synthesized cationic gemini surfactant (SCGS) [16].

Results:

Verification of the SCGS-structure

The chemical structure of the SCGS was verified by FTIR and ^{1}H NMR spectroscopy.

FTIR spectra

FTIR spectra of the SCGS were displayed at bands of 719.39 cm^{-1} (CH rocking), 1368.11 cm^{-1} (CH_3 bending), 1466.49 cm^{-1} (CH_2 bending), 2851.65 cm^{-1} (CH aliphatic asymmetric stretching), 2923.56 cm^{-1} (CH aliphatic symmetric stretching), 1651.03 cm^{-1} (C=N stretching), 1069.41 cm^{-1} (C-N$^+$) and 1586.76 cm^{-1} (C=C stretching) which confirm the expected its functional groups (see Fig. 2) [16].

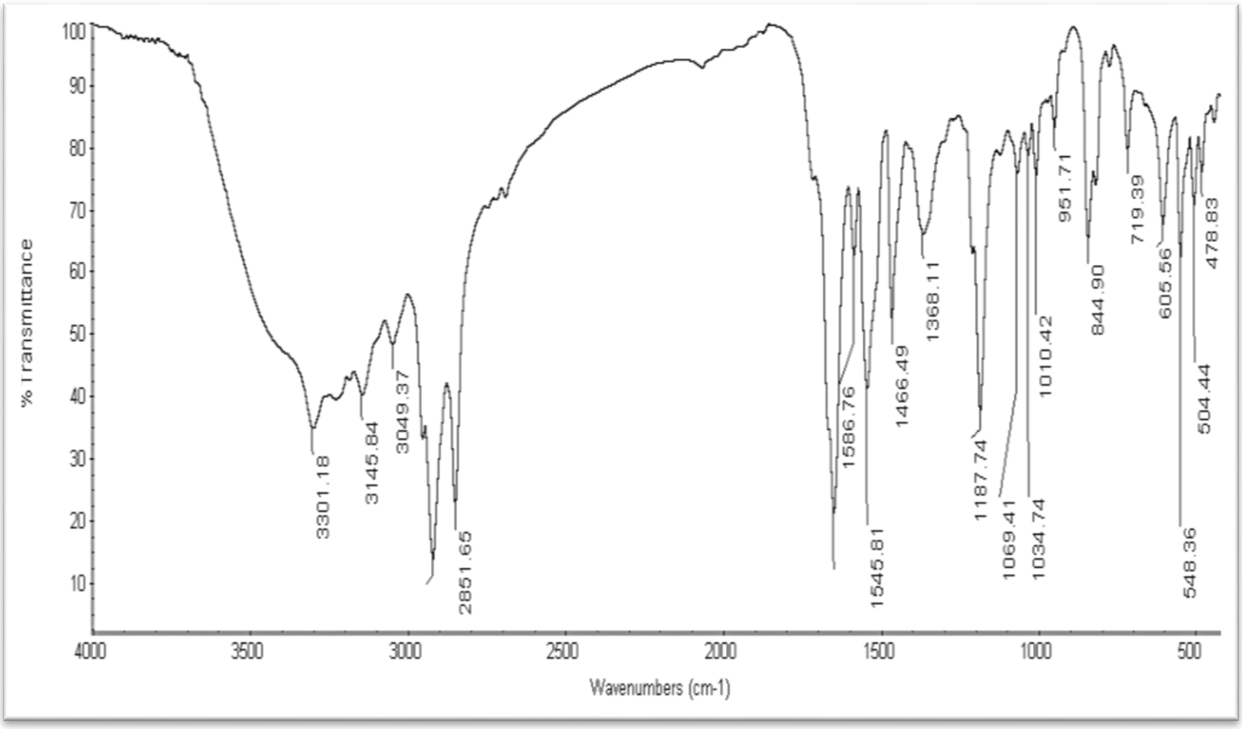

Figure S2. FTIR of the SCGS namely 4,4'-(((1E,5E)-pentane-1,5diylidene)bis (azanylylidene))bis(1-dodecylpyridin-1-ium) bromide [16].

2.1.1. ^{1}HNMR spectra

The ^{1}H-NMR spectra of the SCGS were shown at bands of δ=0.8350-0.0.7947 ppm (t, 6H, $NCH_2CH_2(CH_2)_9C\underline{H}_3$); δ=1.1712 ppm (m, 36H, $NCH_2CH_2(C\underline{H}_2)_9CH_3$); δ=1.6740 ppm (m, 4H, $NCH_2C\underline{H}_2(CH_2)_9CH_3$); δ=4.0806 ppm (m, 4H, $NC\underline{H}_2CH_2(CH_2)_9CH_3$); δ=3.3924 ppm (t, 4H, $NC\underline{H}_2CH_2C\underline{H}_2N$); δ=3.2548 ppm (m, 2H, $NCH_2C\underline{H}_2CH_2N$); δ=8.1425 ppm (d, 4H, m-pyridine); δ=8.2113 ppm (d, 4H, o-pyridine) which confirm its expected hydrogen proton (see Figure 3) [16].

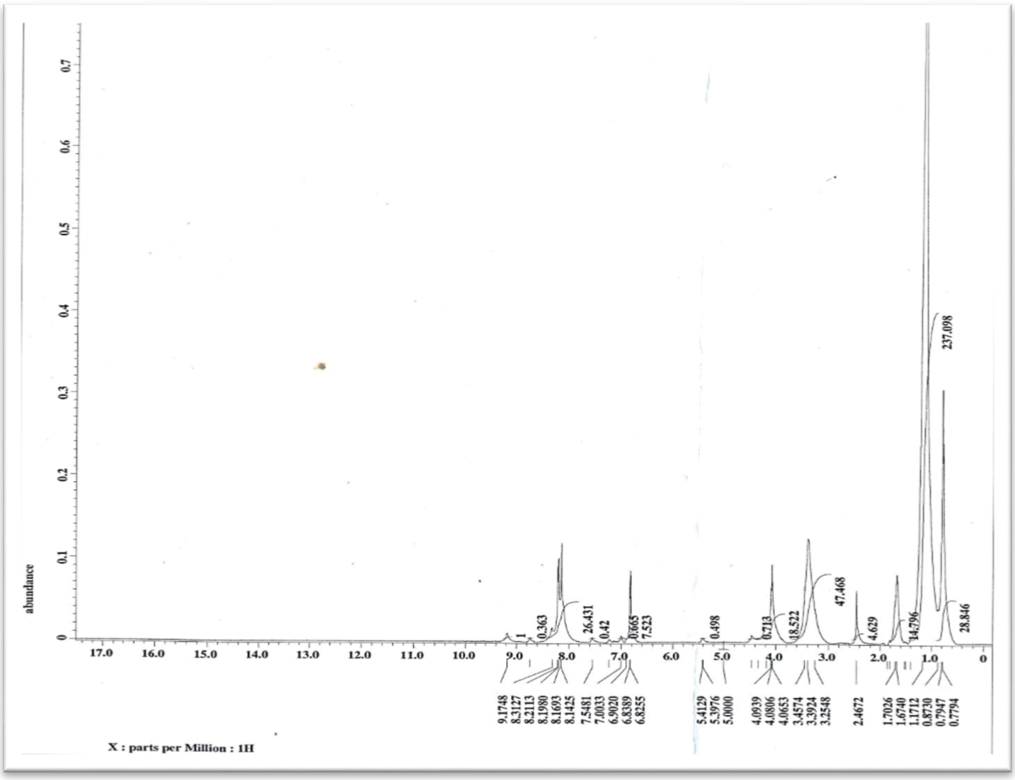

Figure S3. ^{1}H NMR of the SCGS namely 4,4'-(((1E,5E)-pentane-1,5-diylidene) bis(azanylylidene))bis(1-dodecylpyridin-1-ium) bromide [16].

Reference:

M.A. Hegazy, R.M. Samy, A. Labena, M. A.M. Wadaan, W. N. Hozzein. 4,4′-(((1E,5E)-pentane-1,5-diylidene)bis(azanylylidene))bis(1-dodecylpyridin-1-ium) bromide as a novel corrosion inhibitor in an acidic solution (part I), Mater. Sci. & Eng. C, https://doi.org/10.1016/j.msec.2020.110673.
